# Association between cognitive function, antioxidants, and clinical variables in Chinese patients with schizophrenia

**DOI:** 10.1186/s12888-024-06335-5

**Published:** 2024-12-18

**Authors:** Dan Li, Yuanyuan Huang, Hongxin Lu, Sumiao Zhou, Shixuan Feng, Hehua Li, Xuejing Li, Yi Guo, Chunlian Fu, Guiying Chen, Yuping Ning, Fengchun Wu, Lianqi Liu

**Affiliations:** 1 Department of Psychiatry, Guangzhou Civil Affairs Bureau Psychiatric Hospital, Guangzhou, 510430 China; 2 Department of Psychiatry, The Affiliated Brain Hospital, Guangzhou Medical University, Guangzhou, 510370 China; 3 Guangdong Engineering Technology Research Center for Translational Medicine of Mental Disorders, Guangzhou, 510370 China; 4 Key Laboratory of Neurogenetics and Channelopathies of Guangdong Province and the Ministry of Education of China, Guangzhou Medical University, Guangzhou, 510370 China; 5 Department of Psychiatry, Longyan Third Hospital of Fujian Province Department of Psychiatric Medicine, Longyan, Fujian 364030 China

**Keywords:** Cognitive function, Schizophrenia, PANSS, Superoxide dismutase, Serum albumin, Uric acid

## Abstract

**Objective:**

Cognitive dysfunction is a prevalent and intricate manifestation of schizophrenia (SCZ) that may be associated with distinct clinical factors and the presence of antioxidants, which relationship is unclear. The study aimed to investigate cognitive function and its influencing factors in Chinese patients with SCZ.

**Methods:**

A group of 133 patients with SCZ and 120 healthy controls (HCs) were recruited. The MATRICS Consensus Cognitive Battery (MCCB) was utilized to evaluate cognitive ability, and the Positive and Negative Syndrome Scale (PANSS) was used to assess clinical symptoms. Levels of plasma superoxide dismutase (SOD), serum albumin (ALB) and uric acid (UA) were assessed.

**Results:**

Compared with HCs, patients with SCZ exhibited lower cognitive performance as indicated by MCCB scores, including the dimensions of speed of processing, attention/vigilance, working memory, verbal learning, and visual learning. In the SCZ group, total PANSS scores were negatively associated with all MCCB dimensions (all *p* < 0.05), except for the attention/vigilance score. The PANSS-negative and PANSS-cognitive subscores were negatively associated with speed of processing, verbal learning, and visual learning scores (all *p* < 0.05). The PANSS-excited subscores showed a negative correlation with working memory and visual learning scores (all *p* < 0.05). ALB levels significantly decreased, and their UA and SOD levels were notably elevated compared to HCs (all *p* < 0.05). ALB levels and PANSS-negative factors were correlated with to speed of processing, working memory, and visual learning dimensions. SOD levels were independent contributors to the attention/vigilance dimension.

**Conclusion:**

The cognitive function was decreased in SCZ. The degree of cognitive impairment was closely related to ALB, SOD levels and negative clinical symptoms.

## Background

Cognitive dysfunction is a serious and common core symptom in patients with schizophrenia(SCZ), including impairments in attention, learning, executive function, processing speed, language, and memory. Approximately 70-85% of patients with SCZ exhibit cognitive impairment [1, 2], may be subject to duration of illness, age of onset, medication and others, and the degree of cognitive impairment may be related to these factors [3, 4]. Cognitive dysfunction is one explanation for the poor curative effect of current therapies and prognosis of patients, which severely affects social function and the ability to live independently, and even potential impulsive behaviors such as suicide and self-injury [5, 6]. To date, many tools have been used in clinical practice to assess cognitive function in patients with SCZ, many of which rely only on the patient’s medical history interview [7], which is, to some extent, subjectively influenced by the evaluator. Research on the pathophysiology of cognitive decline in SCZ has emphasized issues with neuronal development, disrupted neurotransmission, viral infections, autoimmune problems, and oxidative stress (OS) [8–10]. Recently, there has been an increasing focus on the relationship between SCZ and OS [11], which may provide a direction for exploring objectively effective auxiliary diagnostic techniques and methods for clinical evaluation of cognitive function in individuals with SCZ.

OS is a process in which the body generates an excessive amount of highly reactive molecules upon encountering specific detrimental stimuli. These molecules cannot be completely eliminated by antioxidants, thereby disrupting the delicate balance between oxidation and antioxidant systems [12, 13]. Under normal circumstances, the body possesses a sophisticated antioxidant defense system that uses endogenous antioxidants to effectively eliminate excess free radicals and safeguard tissues from potential damage. Enzymatic and non-enzymatic antioxidants make up the antioxidant defense system [12], including superoxide dismutase (SOD), catalase, and glutathione peroxidase as enzymatic antioxidants, and albumin (ALB), bilirubin, uric acid (UA), vitamin C, and vitamin E as non-enzymatic antioxidants. Various diseases, like tumors, cardiovascular and neurological disorders (e.g., Alzheimer’s disease, Parkinson’s disease, and Down syndrome), as well as mental illnesses such as depression, SCZ, and bipolar disorder, are closely linked to OS [14–16]. Recent research on SCZ and OS revealed a correlation between thioredoxin and cognitive impairments in SCZ patients [17], along with increased levels of nitric oxide in the brains of individuals with SCZ [11, 18]. The majority of past research on OS and cognitive abilities has centered around the topics of physiological senescence and Alzheimer’s disease [19–21]. Further clinical confirmation is necessary to determine the correlation between OS and cognitive performance in individuals with SCZ.

Considering the importance of cognitive function in the rehabilitation and prognosis of patients with SCZ, it is imperative to study its influencing factors and mechanisms, particularly OS. Hardingham et al. [22] found a link between early-life NMDAR dysfunction and OS in SCZ pathogenesis. Studies have shown elevated levels of SOD activity in the blood, red blood cells, prefrontal cortex, and innominate substances in autopsies of individuals with SCZ [23]. Some studies have also indicated reduced SOD1 activity. Multiple studies have indicated that OS plays a role in the development of early cognitive impairment in SCZ [24]. We previously established a correlation between OS markers and cognitive function, gender, and obesity in individuals diagnosed with SCZ, setting the stage for additional research on the link between antioxidant levels and cognitive function in patients with SCZ [25, 26]. Although previous investigations have confirmed the correlation between cognitive function and OS in patients with SCZ, most were laboratory studies. Conveniently assessed blood indicators may be more useful for real-time guidance in clinical practice.

Considering that antioxidants may have a close relationship with cognitive function in SCZ, there are no standardized, consistent, readily available, and affordable oxidative-related biological markers available to indicate cognitive function in individuals with SCZ. This study is the initial investigation into potential factors that could impact cognitive function in SCZ patients through an examination of plasma SOD, as well as serum levels of UA and ALB, and the relationship(s) with cognitive function and clinical manifestations. This provides an objective method for the clinical assessment of cognitive function in patients with SCZ.

## Methods

### Participants

Prior to participating in the research, all participants and their guardians signed an informed consent form. The study was approved by the Ethics Committee of the Affliated BrainHospital of Guangzhou Medical University, approval number: AF/SC-08/02.1. From December 2021 to September 2022, 265 individuals (125 HCs and 140 individuals with SCZ) were enrolled in the study at the Affiliated Brain Hospital of Guangzhou Medical University. Five healthy controls had incomplete information and seven in individuals with SCZ group did not complete cognitive function assessments. Finally 120 HCs and 133 SCZ patients were included in this study. The canteen provides the diet of the inpatients, which is generally made by the dietitian. All the patients had a similar diet and were uniformly provided by the hospital canteen. Each patient in the study, aged 18 to 60, of Han Chinese descent, met all inclusion criteria. They were diagnosed with SCZ according to the Diagnostic and Statistical Manual of Mental Disorders, Fourth Edition (DSM-IV) by two experienced psychiatrists using the Structured Clinical Interview for DSM-IV (SCID-I/P), and had been on a stable dose of antipsychotic medications for at least 8 weeks before the research began. Individuals with significant medical abnormalities, such as unstable or acute medical conditions, comorbid psychiatric disorders, previous history of epilepsy, intellectual disabilities, brain trauma, use of electroconvulsive therapy in the previous six months, and use of any antioxidants or immunomodulators within the last three months were excluded.

Han Chinese 18–60 years of age were recruited through advertisements in a local community in Guangzhou, China. No participants reported a history of psychiatric disorders, either personally or within their family, during the informal interviews. They exhibited comparable dietary patterns, and excluded somatic diseases such as instability or acute illness, as well as epilepsy, mental retardation, and brain trauma, and had not received any antioxidants or immunomodulators within 3 months.

### Clinical measurements

All patients with SCZ and HCs underwent complete medical history taking, laboratory investigations, and physical examinations. General information and sociodemographic characteristics were collected based on questionnaires, and histories of psychiatric disorders and medication use were obtained from medical records by trained research staff. Two trained psychiatrists used the Positive and Negative Syndrome Scale (PANSS) [27] to assess the psychiatric symptoms of all patients with SCZ. The study utilized the PANSS five-factor model to evaluate individuals in 5 different areas: positive symptoms (P1, P5, G9), negative symptoms (N1, N2, N3, N4, N6, G7), excitatory symptoms (P4, P7, G8, G14), depressive and anxious symptoms (G2, G3, G6), as well as cognitive impairments (P2, N5, G11) [28, 29]. The inter-rater reliability coefficient for the PANSS total score surpassed 0.8 in repeated evaluations [30].

### Cognitive assessments

Patients’ cognitive function, such as speed of processing (SOP), attention/vigilance (AV), working memory (WM), verbal learning (VRB), and visual learning (VIS) reasoning and problem-solving, and social cognition, was assessed using the MATRICS Consensus Cognitive Battery (MCCB) tool [31]. The first 5 items assess cognitive function. The domains primarily mirror the cognitive abilities of individuals with SCZ [32]. Research conducted by Shi et al. [33] has shown that the Chinese adaptation of the MCCB exhibits positive psychometric characteristics, suggesting robust reliability and validity.

### Analysis of antioxidants levels

Each participant had venous blood samples collected in the early morning after fasting for at least 8 h. Blood was obtained and placed in an EDTA tube, followed by centrifugation (3000 rpm) for a duration of 10 min within 30 min after collection. A clinical laboratory technician at the hospital performed blood biochemical tests without knowledge of participant information.

Levels of SOD, ALB, and UA were assessed with an automated biochemical analyzer (Beckman AU480, BD Biosciences, Franklin Lakes, NJ, USA) and kits from Beijing Leadman Biotechnology Co., Ltd (Beijing, China). SOD levels were measured using SOD assay kits (enzyme cycle assays). All experimental procedures followed the manufacturer’s instructions meticulously.

### Statistical analysis

The chi-squared test was used to compare demographic variables (i.e., categorical) between patients with SCZ and HCs, while analysis of variance (ANOVA) was used to compare continuous variables. Due to the non-normal distribution of ALB levels (Shapiro–Wilk test, *p* < 0.05), a non-parametric test (Mann–Whitney test) was employed to assess the differences in ALB levels between individuals with SCZ and HCs. ANCOVA was utilized to assess the disparities in levels of UA and SOD, and cognitive performance on the MCCB between the 2 groups after controlling for demographic variables (i.e., gender, age, education) and clinical symptoms (i.e., PANSS total and subscale scores), which revealed significant between-group differences. The relationships among antioxidant enzyme levels, clinical ratings, and cognitive performance were examined using Spearman’s correlation. Furthermore, partial correlation was used to control for additional variables. Multiple tests were adjusted using the Bonferroni correction. Multivariate regression analyses (stepwise regression model) with MCCB subscores were used as dependent variables to investigate the relationship between cognitive function and antioxidant enzyme levels, while controlling for demographic variables and clinical symptoms on the PANSS. Statistical analyses were conducted using SPSS version 18.0 from IBM Corporation in Chicago, IL, USA. Statistically significant differences were defined as those with a two-tailed p-value less than 0.05.

## Results

### Demographic and clinical data

Demographic information and clinical data of the participants included in this study are summarized in Table 1. There were significant differences in marital status, education, and body mass index (BMI) between the 2 groups (all *p* < 0.05); however, there were no significant disparities in terms of gender or age. Gender, age, education BMI were adjusted for in subsequent analyses.

**Table 1:** Demographics of SCZ patients and HCs

Variable	SCZ patients n = 133	HCs n = 120	F/χ2	*p*-value
Age (years)	44.74 ± 12.84	43.52 ± 13.06	0.560	0.455
Male/female, n	77/56	63/57	0.743	0.448
Married status			52.774	< 0.001
Married	22(16.5%)	73(60.8%)	-	-
Unmarried	111(83.5%)	47(39.2%)	-	-
Education (years)	11.08 ± 3.36	14.08 ± 3.35	50.089	< 0.001
Body mass index (BMI) (kg/m^2^)	24.15 ± 4.28	23.08 ± 3.85	4.892	0.028
Family history, n(%)	31(23.3%)	-	-	-
Age of onset (years)	25.26 ± 7.43	-	-	-
Duration of disease (months)	220.41 ± 157.73	-	-	-
Antipsychotic dosage (mg/day), CPZ equivalents	406.63 ± 180.06	-	-	-
PANSS total score	58.24 ± 18.86	-	-	-
Positive subscore	6.91 ± 3.69	-	-	-
Negative subscore	14.62 ± 7.57	-	-	-
Excited subscore	5.29 ± 2.17	-	-	-
Depressive subscore	6.16 ± 3.15	-	-	-
Cognitive subscore	4.77 ± 2.39	-	-	-

PANSS: Positive and Negative Syndrome Scale; *p* < 0.05,the difference was statistically significant

### Cognitive function of patients with SCZ and HCs

Table 2 summarizes information on cognitive function (MCCB performance) in individuals with SCZ and HCs. Compared with HCs, patients with SCZ exhibited worse cognitive performance in terms of SOP, AV, WM, VRB, and VIS. The significant differences persisted even after adjusting for gender, age, education, BMI level (all *p* < 0.05). In the SCZ group, the PANSS total scores were negatively associated with all MCCB indices (all *p* < 0.05) except AV. The PANSS-negative and PANSS-cognitive subscores had a negatively correlation with the SOP, VRB, and VIS scores (all *p* < 0.05). There was a negative correlation between PANSS-excited subscores and WM and VIS scores (all *p* < 0.05).

**Table 2:** Cognitive functions (MCCB) of SCZ patients and HCs

Variable	SCZ patients n = 133	HCs n = 120	F	*p*-value	Adjusted F*	Adjusted p*	
SOP score	31.01 ± 17.18	50.99 ± 9.17	129.180	< 0.001	67.016	< 0.001	
AV score	36.55 ± 10.71	49.61 ± 9.89	100.885	< 0.001	60.837	< 0.001	
WM score	35.65 ± 13.74	48.89 ± 9.63	77.259	< 0.001	28.888	< 0.001	
VRB score	32.29 ± 12.78	45.11 ± 10.45	75.363	< 0.001	44.997	< 0.001	
VIS score	34.94 ± 12.19	48.34 ± 10.62	86.292	< 0.001	47.801	< 0.001	

*Adjusted values were calculated with gender, age, education, BMI as covariates. MCCB, the MATRICS Consensus Cognitive Battery of tests; SOP, speed of processing; AV, attention/vigilance; WM, working memory; VRB, verbal learning and memory; VIS, visual learning and memory

### ALB, UA, and SOD levels in patients with SCZ and HCs

**Fig. 1:**
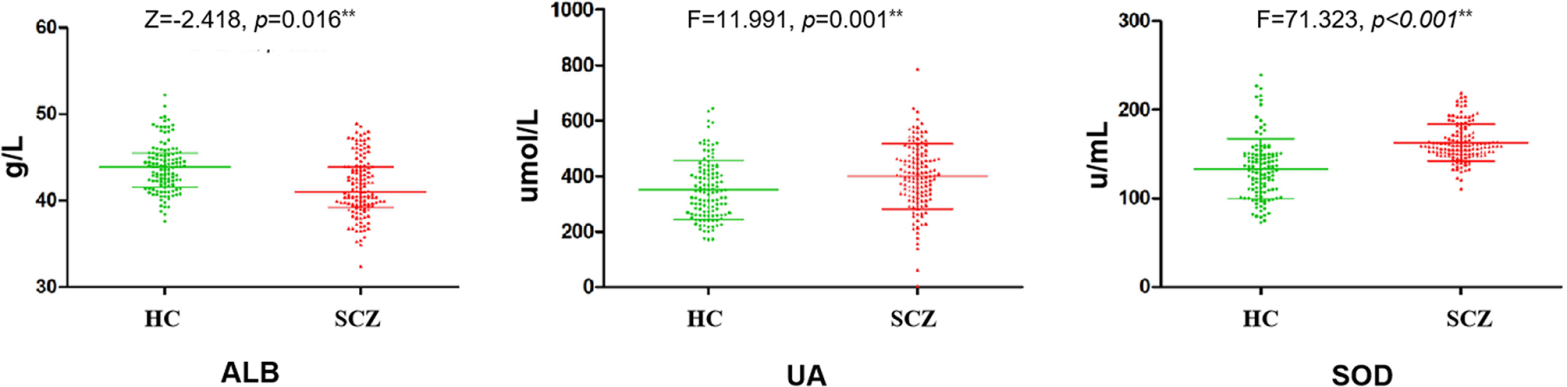
The levels of antioxidants between SCZ patients and HCs

Antioxidant levels (ALB, UA, and SOD) in individuals with SCZ and HCs are presented in Fig. 1. Serum ALB levels were significantly lower in those with SCZ (median 42.45 ([P25, P75] 39.82, 46.10]) than in HCs (median 44.52 [41.55, 48.48]; z = -2.418; *p* = 0.016). Even after accounting for gender, age, education, BMI, the distinction in ALB levels between the two groups continued to be noteworthy (*p* < 0.05).

Elevated levels of Serum UA and SOD were observed in participants diagnosed with SCZ when compared to HCs (400.16 ± 117.63 versus [vs.] 351.28 ± 105.48 µmol/L; F = 11.991, *p* = 0.001; 162.94 ± 21.00 vs. 133.22 ± 33.91 U/mL; F = 71.323, *p* < 0.001). These variations remained significant even after accounting for factors such as gender, age, education, BMI (F = 8.613, *p* = 0.004; F = 33.328, *p* < 0.001).

Levels of antioxidants did not show any correlation with demographic factors in the two groups (all *p* > 0.05), except ALB and SOD levels, which were inversely related to age in both the HCs and SCZ groups (*p* < 0.05). Within the SCZ cohort, the levels of ALB and SOD showed an inverse correlation with the duration of the disease, while the levels of UA exhibited a positive correlation with PANSS-negative factors.

**Fig. 2:**
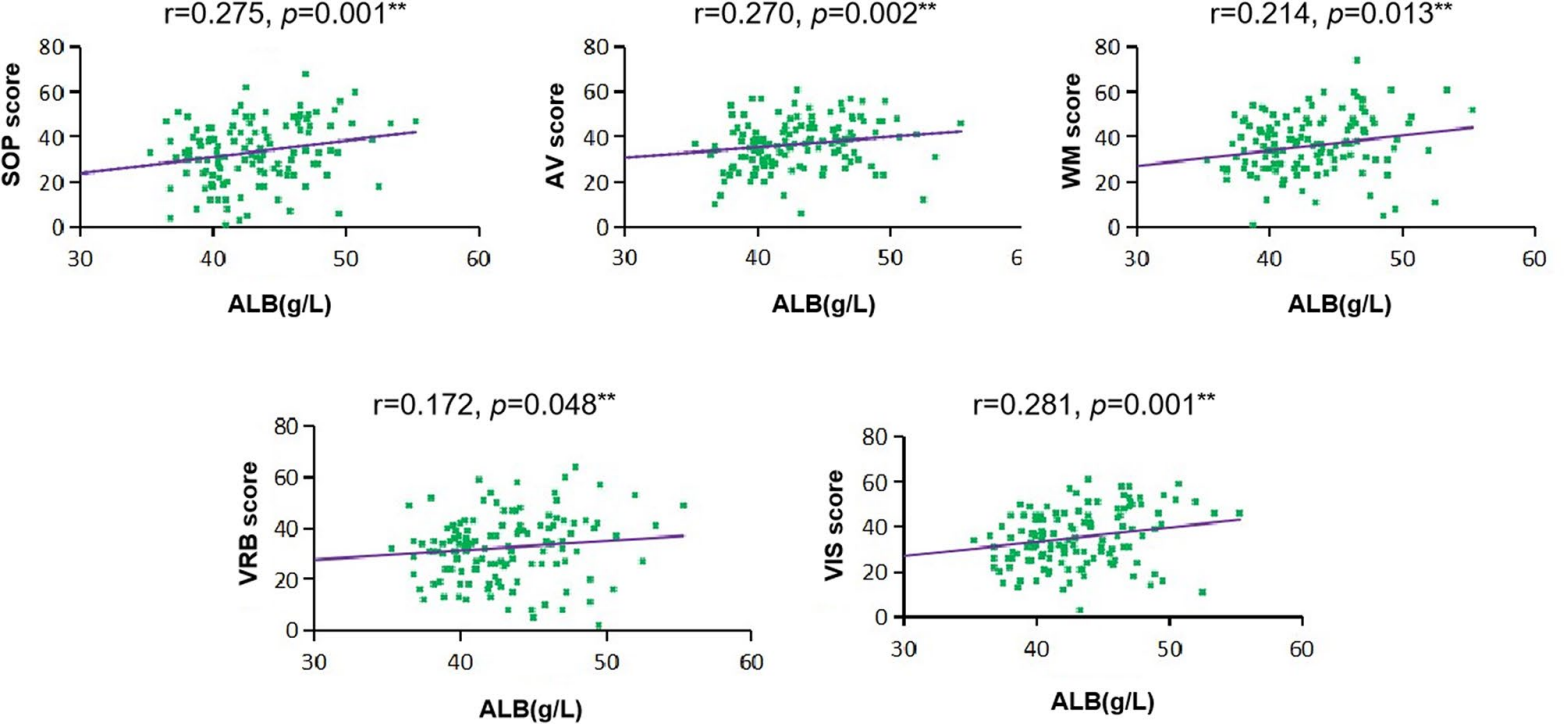
Correlation between cognitive performance and ALB levels in SCZ patients

Spearman correlation analysis revealed that antioxidant levels were associated with partial cognitive function in the SCZ group (Table 3). Within the SCZ group, there was a positive correlation between ALB levels and cognitive performance across 5 MCCB indexes (*r* = 0.172–0.281, *p* < 0.05) (Fig. 2). After adjusting for gender, age, education, BMI, partial correlation analysis reaffirmed the strong connection (*r* = 0.177–0.292, *p* < 0.05). All correlations passed Bonferroni correction (*p* < 0.05); however, the association between VRB score and ALB level failed (*p* > 0.05).

Cognitive performance showed a positive correlation with SOD levels (*r* = 0.183–0.222, *p* < 0.05), except VRB score (*p* > 0.05). After adjusting for gender, age, education, BMI, the partial correlation analysis provided additional evidence of a link between SOD levels and AV (*r* = 0.299, *p* = 0.001; Bonferroni-corrected *p* < 0.05) as well as VIS scores (*r* = 0.217, *p* = 0.014; Bonferroni-corrected *p* > 0.05). There was no correlation between the cognitive performance of the HCs and the levels of the 3 antioxidants (all *p* > 0.05). Levels of UA were not found to be correlated with cognitive performance in SCZ.

**Table 3:** Correlation between five dimensions MCCB and ALB, UA and SOD in patients with SCZ

		SOP score	AV score	WM score	VRB score	VIS score
ALB(g/L)	r	0.275	0.270	0.214	0.172	0.281
	*p*	0.001	0.002	0.013	0.048	0.001
SOD(U/ml)	r	0.183	0.183	0.183	0.132	0.200
	*p*	0.035	0.035	0.035	0.130	0.021
UA(umol/L)	r	-0.013	0.032	-0.023	-0.031	0.041
	*p*	0.882	0.717	0.791	0.722	0.642

Additionally, a step-by-step multiple regression analysis was conducted to elucidate how antioxidants, demographic factors, and clinical manifestations impact the cognitive abilities of individuals diagnosed with SCZ. As shown in Table 4, ALB levels and PANSS-negative factor were independent contributors to the SOP, WM, and VIS indices. SOD levels (beta = 0.14, t = 3.262, *p* = 0.001) were independent contributors to the AV index.

**Table 4:** Factors for cognitive performance in SCZ patients

Dependent variable		B	S.E	t	*p*-value	95% CI	
						Lower	Upper
SOP score	(constant)	-10.946	11.655	-0.939	0.349	-34.003	12.112
	ALB(g/L)	1.164	0.270	4.307	< 0.001	0.629	1.698
	PANSS-negative factor	-0.524	0.183	-2.856	0.005	-0.887	-0.161
AV score	(constant)	13.779	7.037	1.958	0.052	-0.141	27.699
	SOD(U/ml)	0.14	0.043	3.262	0.001	0.055	0.224
WM score	(constant)	9.607	9.673	0.993	0.322	-9.529	28.744
	ALB(g/L)	0.72	0.224	3.211	0.002	0.277	1.164
	PANSS-negative factor	-0.319	0.152	-2.096	0.038	-0.62	-0.018
VIS score	(constant)	10.59	8.467	1.251	0.213	-6.16	27.34
	ALB(g/L)	0.686	0.196	3.494	0.001	0.297	1.074
	PANSS-negative factor	-0.334	0.133	-2.509	0.013	-0.598	-0.071

Variables in the model: gender, age, education, BMI, PANSS total scores and its subscores, ALB, UA, and SOD.

## Discussion

This research was the initial exploration into the correlation between enzymatic and non-enzymatic antioxidants (SOD, ALB, and UA) and the cognitive function and symptoms of individuals diagnosed with SCZ. The primary discoveries of this research were the following. First, patients with SCZ exhibited extensive cognitive impairment compared with HCs. Second, PANSS-negative sub-scores showed a negative correlation with SOP, WM, and VIS scores. Third, SOD levels were positively correlated with cognitive performance (except VRB) in the SCZ group, which was an independent contributor to the AV index. Fourth, ALB levels in the SCZ group were independent contributors to the SOP, WM, and VIS indices. Patients with SCZ had notably elevated UA levels compared to HCs, however, these levels did not show a correlation with cognitive function. These results indicate that different antioxidant enzymes may have close relationships to different cognitive dimensions in patients with SCZ.

Our study found that individuals diagnosed with SCZ exhibited significant cognitive deficits in SOP, AV, WM, VRB and VIS, indicating widespread cognitive impairment, aligning with findings from earlier research studies [1, 34–37]. Cognitive impairment may impact the daily activities of individuals with SCZ, leading to a suboptimal treatment response, challenges in functional recovery, and an elevated risk for long-term disability. The causes of cognitive decline in SCZ are intricate, with growing proof suggesting a common disease-causing gene, epigenetic control of DNA, and resulting changes in the proteome and metabolome, potentially impacting cognitive abilities [38, 39]. The neurobiological basis of cognitive impairment commonly involves reduced levels of gray matter neurons, abnormal myelin density, and white matter cellulose connectivity [40], impaired signal integration at the neuronal and neural network levels, neurotransmitter abnormalities, immune dysregulation, and OS [34, 41, 42]. We explored the relationship between antioxidant levels and cognitive function in patients with stable SCZ, suggesting the need for future research in first-episode SCZ patients and those who have not yet received medication.

In the SCZ group, all MCCB indices showed a strong inverse relationship with the total PANSS score, except AV. Moreover, the adverse factor rating of the PANSS showed a notable inverse correlation with scores for SOP, WM, and VIS, indicating that increased severity of negative symptoms in individuals with SCZ is linked to decreased cognitive abilities, especially in relation to SOP, WM, and VIS. Consequently, it can be deduced that reducing negative symptoms may improve the cognitive function of patients with SCZ. Negative symptoms are a primary factor contributing to disability in individuals with SCZ [5, 43]. A meta-analysis of 21 studies concluded that negative symptoms were associated with neurocognitive function [31], even in high-risk groups [44], aligning with our own findings and may be related to their common neurobiological mechanisms. Disruption of connectivity or decrease in network functional connectivity between the cerebellum and prefrontal cortex and defects in the glutathione system may be related to negative symptoms and cognitive deficits [45, 46]. After stepwise multiple regression analysis, we found that PANSS negative factors independently contributed to the SOP, WM, and VIS indices. Negative symptoms and cognitive deficits may be associated with disruptions in connectivity or reduced functional connectivity between the prefrontal cortex and cerebellum, as well as abnormalities in the glutathione system [47, 48].

Our research found that levels of SOD were significantly higher in the SCZ group than in HCs. Most previous studies have also reported that SOD levels in patients with chronic SCZ exceeded normal levels [49, 50], while others have found no significant change in SOD levels [51] or decreased manganese SOD levels [52]. The inconsistent findings may be explained by the different types of samples (such as cerebrospinal fluid, red blood cells, serum) and differences in SOD determination methods, which may have affected test results.Notably, the SOD levels of patients with SCZ were positively correlated with cognitive performance (exceptVRB). Despite this, there was not a notable correlation between the amounts of these 3 antioxidants and cognitive abilities in HCs, suggesting a robust connection between antioxidants and cognitive decline in individuals with SCZ. Serum antioxidants are associated with cognitive pathophysiology in patients [53, 54], and SOD is a specific enzyme that “cleans” free radicals and protects the body [55]. Stepwise multiple regression analysis revealed that SOD level was an independent contributor to the AV index, suggesting that the higher the SOD level, the higher the AV of patients, and the serum SOD level may predict the cognitive level of patients. Our findings are in line with previous research. Lin et al. found that higher SOD levels were associated with better SOP, WM, and VRB in chronic SCZ [3], aligning somewhat with our findings, possibly attributed to the neuroprotective properties of antioxidants on neurons [56]. Interestingly, Li et al.found that the correlation between overall antioxidant levels and cognitive abilities in patients could be affected by age [3]. We also found that SOD levels decreased with increasing disease duration, suggesting that this may be related to age. Hence, our discovery that levels of antioxidants are linked to cognitive abilities in individuals with SCZ may provide a reference for the clinical search for objective markers of cognitive impairment and a basis for clinical interventions. Drugs that can be used early in the clinic, or dietary or behavioral interventions that can affect antioxidant levels, may lead to better cognitive function or, at least, less significant cognitive impairment.

A noteworthy discovery from this research was that levels of ALB in the serum were notably reduced in individuals with SCZ compared to controls, and were inversely linked to the length of illness and positively linked to cognitive performance on MCCB (excluding VRB). Prior research has consistently shown results that align with our study, suggesting that a decrease in ALB levels in individuals with SCZ is linked to the advancement of the disease [57]. This may be attributed to the inhibitory effect of ALB on lipid peroxidation and its direct removal of oxygen free radicals [58]. The drop in serum ALB levels in individuals with SCZ may be due to the rise in OS damage and antioxidant usage. Moreover, ALB level was correlated with age in all populations, and the slow decline with age may be a natural result of the aging process. Another possible contributing factor is that poor diet may lower ALB levels [59, 60]. The diets of the two groups in this study were essentially the same, which was provided by the hospital. Interestingly, ALB levels remained positively correlated with cognitive performance on the 5 MCCB measures (except VRB) in the SCZ group after removing confounding factors and were an independent contributor to the SOP, WM, and VIS indices. We speculated that ALB levels may be predictive of cognitive function in patients. As simple, convenient, and economical routine clinical examination items, the determination of ALB and SOD levels is undoubtedly a great advantage in clinical applications. If they have the potential to be utilized for an objective assessment of cognitive function in individuals diagnosed with SCZ. Further longitudinal prospective studies with larger sample sizes are required to confirm the role of ALB level in predicting cognitive function in patients with psychiatric disorders.

The study revealed that serum UA levels in SCZ patients remained significantly elevated compared to HCs even after controlling for confounding factors and were found to be positively associated with PANSS-negative factors. Most studies reported similar findings [61]. However, the association between UA levels and SCZ has been a subject of debate in the literature. Based on research investigating SCZ and schizoaffective disorder, and bipolar or depressive disorder, it was found that UA level was decreased in patients with SCZ, while it was unchanged in other diseases [57]. The heterogeneity of the findings may be due to the limitation of the sample size or confounding factors, such as diet, smoking, and medication. As a simple and easily available laboratory indicator, the relationship between UA and the symptoms and cognition of those with mental disorders merits further exploration. Regrettably, there was no notable correlation discovered between serum UA levels and cognitive performance in individuals with SCZ. Collectively, our findings suggest that different antioxidant enzymes have different effects on cognitive function, providing a direction for future research.

### Limitations and future research directions

This study had several limitations, the first of which was its cross-sectional design, and because redox regulation is dynamic, changes occurred at different stages of disease, as such, longer observation periods are necessary. Second, we only included patients with stable SCZ, excluding those with first-episode SCZ or unmedicated patients, and did not limit the type or dosage of medication. Further research is required in this area. Third, diet tended to influence SOD, UA, and ALB levels. The diets of the patients included in the present investigation were provided by the hospital cafeteria during their hospitalization and were approximately the same, but the amount of food eaten and some snacks are not strictly limited controlled. Fourth, smoking, unprescribed psychological consultation or therapy, etc. may affect oxidative stress or cognition, which has not been recorded and analyzed, and we will further study it in subsequent research.

## Conclusions

In conclusion, our research demonstrated that patients with SCZ exhibited extensive cognitive functional impairment. The severity of cognitive function impairment is closely associated with ALB and SOD levels as well as negative symptoms. ALB and SOD levels are stable, easily obtainable, and cost-effective biomarkers for the early identification and intervention in patients with SCZ. However, this cross-sectional study only established an association between cognitive impairment and antioxidants rather than causation. In follow-up studies, large-sample prospective studies with strict control of disease course, drugs, and other factors can better illustrate the role of antioxidant enzyme levels in the cognitive function of individuals diagnosed with SCZ and provide objective evidence supporting clinical evaluation and early intervention.

## Data Availability

The datasets generated during the current study are not publicly, but are available from the corresponding author on reasonable request.
